# Pathological Left Ventricular Hypertrophy and Stem Cells: Current Evidence and New Perspectives

**DOI:** 10.1155/2016/5720758

**Published:** 2015-12-20

**Authors:** Maria E. Marketou, Fragiskos Parthenakis, Panos E. Vardas

**Affiliations:** Cardiology Department, Heraklion University Hospital, P.O. Box 1352, 711 10 Crete, Greece

## Abstract

Left ventricular hypertrophy (LVH) is a strong predictor of adverse cardiovascular outcomes. It is the result of complex mechanisms that include not only an increase in protein synthesis and cell size but also proliferating cardiac progenitor cells and the influx of bone marrow-derived cells developing into cardiomyocytes. Stem and progenitor cells are known to contribute to the renewal of adult mammalian cardiomyocytes in case of myocardial injury or pressure and volume overload. They are activated in LVH and play a regulatory role in myocardial repair. They have high proliferative potential and secrete numerous cytokines, growth factors, and microRNAs that play important roles in cell differentiation, cardiac remodeling, and neovascularization. They are mobilized in response to either mechanical or chemical stimuli, hormones, or pharmacologic agents. Another important source of progenitor cells is the epicardial layer. It appears that precursor cells migrate from the epicardium to the myocardium in order to interact with myocardial cells. In addition, migratory cells participate in the formation of almost all cardiac structures in myocardial hypertrophy. Although the pathophysiological mechanisms are still obscure and further studies are required, their properties may open the door to regenerative cell therapy for the prevention of adverse remodeling.

## 1. Introduction

Left ventricular hypertrophy (LVH) is a strong predictor of adverse cardiovascular outcomes and an important risk factor for sudden death and heart failure [1]. LVH is a complex and multifactorial condition whose pathogenesis may include many different genetic and signaling pathways [2]. It involves a process of adaptive remodeling, which is usually a compensatory mechanism in response to increased hemodynamic load. Initially, this mechanism is beneficial in most cases [2]. However, ultimately it is characterized by structural changes, mainly in the form of myocardial fibrosis, that lead to diastolic dysfunction and diminished contractility.

## 2. Physiological and Pathological Myocardial Hypertrophy

In general, LVH is represented by physiological and pathological myocardial hypertrophy. Physiological cardiac hypertrophy occurs in two basic settings: exercise training and pregnancy. Cardiac hypertrophy may be a “physiological” adaptation when it occurs in healthy conditions, such as in athletes or in pregnant women. During normal development, the heart grows after birth, increasing both the size and the number of cardiomyocytes together with other cellular structures, such as the vasculature net. Hypertrophy, even of the physiological type, results not only from the growth of preexisting myocytes but also from an increase in their number via the formation of new cardiomyocytes [3]. Cardiac progenitor cells participate in this hypertrophy, as can be seen from their mobilization and activation during exercise [4–6]. Animal studies have indicated that activation of c-kit^+^ endogenous cardiac stem cells (CSCs), which increase in number and undergo a process of cell specification and differentiation towards the myocyte and vascular lineages, accompanied by increased levels of growth factors, is key feature of physiological myocardial hypertrophy [6]. CSCs include, among others, c-kit^+^Lin^−^ and Sca-1^+^-Lin^−^ cells, Isl-1 progenitors, epicardial progenitors, and progenitors generating cardiospheres and muscle-derived mesenchymal stem cells. Experimental studies have shown that it is mostly c-kit^+^Lin^−^ cells that seem to increase in the heart in response to exercise training [5]. However, it has been speculated that Sca-1^+^-Lin^−^ might also be stimulated in physiological hypertrophy. It should be emphasized that our data regarding physiological hypertrophy and stem cells originated from studies of exercise training, since there are limited data for pregnancy and the postnatal heart.

In all other situations, after the initial compensatory and adaptive phase, hypertrophy can lead to a decline in ventricular function and finally to heart failure. The mechanisms of pathological cardiac hypertrophy include those mentioned above but are more complex and vary from case to case. One of the most common causes is pressure overload, which leads to an increase in wall thickness and concentric hypertrophy of the left ventricle as a compensatory mechanism to maintain the ventricular ejection fraction under conditions of increased peripheral resistance. Recent studies in mammalian hearts have also found increased numbers of c-kit^+^ cells in pressure overload conditions that lead to congestive heart failure [7]. However, it has been reported that c-kit-expressing cardiac progenitor cells are usually the primary source for the generation of cardiac endothelial cells, rather than cardiomyocytes [8].

Another mechanism involves volume overload, in conditions such as chronic aortic regurgitation, mitral regurgitation, or anemia, which lead to lengthening of myocardial fibers by sarcomere replication in series and an increase in ventricular volume. This pattern of eccentric hypertrophy is also initially compensatory, so that the heart can meet the demand to sustain a high stroke volume. It should be noted that concentric hypertrophy may progress to eccentric, while chronic hypertrophy in general may be detrimental, resulting in most cases in heart failure and cardiovascular death. Hypertrophy is usually accompanied by complex gene reprogramming in the cardiac cell population [9]. The expression of the fetal genes *α*-skeletal actin and *β*-myosin heavy chain is believed to be the cornerstone in the pathophysiology of pathological hypertrophy [10].

An increased cardiac workload in pathological situations often initiates a cascade of biological events that lead to cardiomyocyte hypertrophy and increased cardiac growth [11]. A feature of LVH is that it entails an increase in the number of sarcomeres, preceded by biomechanical signal transduction alterations in growth and myocyte-extracellular matrix coupling for force generation [12, 13]. It is also accompanied by a proportional increase in perivascular and interstitial connective tissue and ground substance, as well as in the capillary and nerve networks [14]. On the one hand, the physiological hypertrophied heart maintains the normal cardiac structure. On the other hand, left ventricular remodeling and hypertrophy during increased cardiac load result in contractile dysfunction and heart failure, which are associated with high morbidity and mortality [1].

Many aspects of the pathophysiological mechanisms involved in this process are still unclear and incompletely understood. In particular, the differential role of stem cells in the adaptive and potentially reversible physiological hypertrophy compared to the pathological form is still obscure. It is likely that neurohumoral activation and stimulation of certain growth factors may play a key role. Further study and a better understanding of the pathogenetic role of stem cells in LVH could lead to the development of targeted therapeutic interventions for prevention or reversal of the phenomenon.

## 3. Myocardial Regeneration and Stem Cells

LVH is the result not only of an increase in protein synthesis and cell size but also of proliferating cardiac progenitor cells and the influx of bone marrow-derived cells developing into cardiomyocytes [3–6]. The discovery that there are stem cells in the heart that can differentiate into the various cardiac cell lineages necessitated a reconsideration of the mechanisms of myocardial pathophysiology and in particular of the progression into cardiac hypertrophy. Stem cells have a high capacity for cell division [6]. They have the potential for self-renewal and differentiation and are the progenitor cells of various mature cells [6]. Certain stem cells may have a greater capacity to transdifferentiate, whereas others may show greater paracrine activity or a greater potential to stimulate neovascularization. CSCs can regulate myocyte turnover and myocardial recovery after injury [15]. Under pathological conditions and in myocardial injury, it appears that, apart from CSCs, bone marrow-derived stem cells are also mobilized, as can be seen from changes in the number and function of circulating progenitor cells. These have been found to show different behavior in many cardiovascular diseases, although the associated pathophysiology has not yet been fully elucidated. The most characteristic example is following acute myocardial infarction, when the numbers of circulating CD34^+^ and CD133^+^KDR^+^ endothelial progenitor cells are upregulated in response to tissue ischemia [16].

In particular, in the presence of pressure and volume overload, progenitor cells are recruited in the myocardium and lead to hypertrophy, a process that is guided by the SDF-1/CXCR4 axis [17]. Studies of posttransplant organs have shown that there is a circulating pool of stem cells that participate in the regeneration of myocardium [18]. At least 2% to 5% of chimeric myocytes have been detected in some studies, confirming that blood-borne cardiac-committed cells may reach the myocardium [19, 20].

It used to be the view that human cardiomyocytes had no renewal capability and exhibited very limited regenerative capacity. However, that view has been revised, since studies have shown that approximately 1% of cardiomyocytes are renewed per year at age of 20 years and 0.4% at age of 75 years [21]. On that basis, about 45% of cardiomyocytes would be renewed over the normal human lifespan. There is evidence of myocyte renewal in the adult heart, which is capable of limited regeneration from cardiac-endogenous precursors [21].

The human heart is in continuous turnover and has an intrinsic regenerative potential, which is based on a portion of the reservoir of stem and progenitor cells that exists in myocardial tissue. These cells are positive for various stem cell markers, such as c-kit, MDR-1, and stem cell antigen-1 [18].

This ability of the myocardium to regenerate myocardial cells under physiological conditions, as well as to compensate for pathological disturbances, is due, by a large degree, to the existence of cardiac progenitor cells in postnatal hearts [15]. Stem cells are of great importance in postnatal life, owing to their ability to replace senescent cells and regenerate damaged organs [22]. CSCs have been proved to exist in the human heart and can differentiate into the various cardiac cell lineages. They can regulate myocyte regeneration after injury or mechanical stress.

CSCs are stored in niches, which are the microenvironment within which stem cells remain in their undifferentiated state or, under special conditions, receive growth signals from other cells. This may trigger the growth, migration, and commitment of CSCs that leave the niches and home into myocardial tissue [23].

Another important source of progenitor cells is the epicardial layer. The epicardial marker WT1 appears to regulate the epithelial-mesenchymal transition [24] and WT1-positive progenitors migrate from the proepicardium to the myocardium in order to interact with myocardial cells [25]. Moreover, a population of proepicardial Tbx18-positive progenitors may give rise to a substantial fraction of cardiomyocytes [26]. An important role in the differentiation of these cells into myocardial cells is played by connexin 43, which also promotes electric coupling in cells committed to the myocyte phenotype.

However, the precise origin of cardiac progenitor cells is still unclear. One possible scenario is that they are also recruited from bone marrow; however, the data in LVH are limited and further studies are needed to determine the precise mechanisms. Progenitor cells have high proliferative potential and secrete numerous cytokines, growth factors, and microRNAs (miRNAs) that play important roles in cell differentiation, cardiac remodeling, and neovascularization [27, 28]. Duran et al. demonstrated that bone marrow-derived stem cells improve survival and cardiac function and attenuate remodeling through the secretion of proangiogenic factors that stimulate endogenous neovascularization and differentiation into functional adult myocytes and vascular cells [29].

Numerous studies have confirmed that, in other cardiovascular conditions, such as myocardial infarction, there is a rapid mobilization of hematopoietic stem cells: endothelial progenitor cells (EPCs) and mesenchymal stem cells (MSCs) [16, 30, 31]. In addition, it has already been proved that stem cells are implicated in ventricular remodeling [32] and play a major role in the homeostasis of the heart [33], as well as in the pathophysiology of heart failure [34].

Among the stem and progenitor cells, EPCs are very important and make a major contribution to postnatal physiological and pathological neovascularization, while they are an attractive field for research, as they have been proven to participate in the regeneration of injured endothelium. Previous studies have shown that EPCs originating from CD-34^+^ cells in peripheral blood may participate in vasculogenesis in animal hindlimb ischemic models [35]. These EPCs have limited proliferating potential for long-term culture. On the other hand, EPCs originating from bone marrow showed different properties [36].

EPCs are a heterogeneous population in terms of lineage, phenotype, and growth pattern, and it is difficult to fully determine their precise role. Although they are generally defined as cells that express a variety of cell surface markers, similar to those expressed by vascular endothelial cells, many different EPC definitions have been used. Hur et al. cultured total mononuclear cells from human peripheral blood to obtain two types of EPC sequentially from the same donors. The authors found two types of EPC from a single source of human peripheral blood: early and late EPCs [37]. Early EPCs showed a spindle shape, had peak growth at 2 to 3 weeks, and died at 4 weeks. Late EPCs, with a cobblestone shape, appeared late at 2 to 3 weeks, showed exponential growth at 4 to 8 weeks, and lived up to 12 weeks. Late EPCs were different from early EPCs in the expression of VE-cadherin, Flt-1, KDR, and CD45. Late EPCs produced more nitric oxide (NO) and formed capillary tubes better than early EPCs. Early EPCs secreted more angiogenic cytokines than did late EPCs during culture in vitro. Both types of EPC showed comparable in vivo vasculogenic capacity.

Some studies have shown that circulating EPCs play a key role in the maintenance of endothelial homeostasis and promote vascular repair [38]. The bone marrow-derived EPCs migrate to the injured endothelium and differentiate into mature endothelial cells in situ [39]. Published data have proved that EPCs are mobilized from the bone marrow in response to injury of the endothelium and that the recruitment of circulating EPCs is critical for the vascular remodeling and repair process [40].

Apart from hypertrophy and the proliferation of cardiomyocytes, there is also an increase in vasculature, which occurs via various mechanisms, including vasculogenesis through neovascularization, which is mediated by the migration of EPCs from the bone marrow [41].

**Figure 1:**
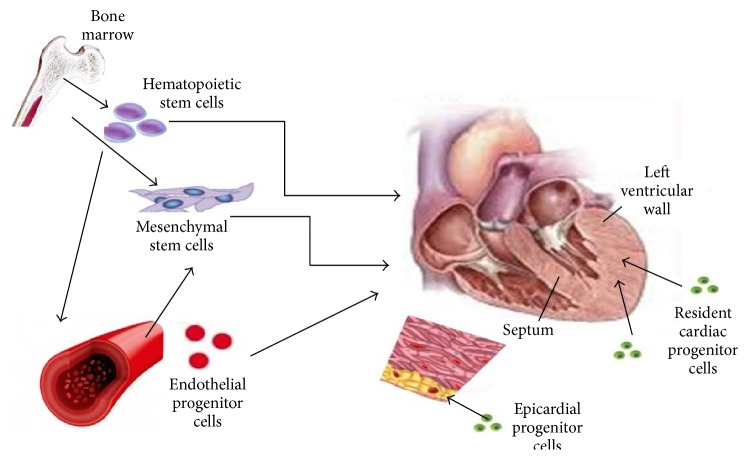
Sources of progenitor cells that participate in myocardial regeneration and remodeling in left ventricular hypertrophy.

However, the mobilization of stem cells into peripheral blood is not sufficient in itself; homing, adhesion, and engraftment of these cells onto the cardiac tissue are also required. This process requires signaling mediated by chemokines, which are important regulators of cell trafficking and function. These cells can be mobilized in response to various stimuli, either mechanical or chemical, hormones, and angiogenesis-promoting growth factors [41, 42]. It is of interest that pharmacological agents, such as those that act on the renin-angiotensin system, might regulate EPC activation, adding therapeutic dimensions to the phenomenon and perhaps partially explaining their beneficial action in LVH regression [43]. Failure of the heart to adapt to pathological loads may reflect the lack of translation of mechanical signals from the organ to human CSC (hCSC) niches, or the demand for regeneration of SMCs, ECs, and myocytes may be so high that myocardial niches become depleted of hCSCs. The latter possibility may result in the formation of empty dysfunctional niches or niches in which resident hCSCs have reached irreversible growth arrest and cellular senescence (Figure 1). Depletion of functional hCSCs may severely decrease the generation of vascular cells and cardiomyocytes, resulting in excessive myocyte hypertrophy and impaired coronary perfusion. Whether the delivery of functionally competent hCSCs repopulates empty niches, regenerates the scarred myocardium, reverses the cardiac phenotype, and rescues the failing heart remains the major challenge of stem cell therapy.

The phenomenon of mobilization and activation of bone marrow-derived stem cells in response to cardiac pressure overload is attenuated with aging [44]. Older bone marrow is associated with decreased cardiac function, increased fibrosis, and decreased myocyte hypertrophy and bone marrow cell engraftment in the myocardium [44].

## 4. Description and Location of CSCs

CSCs are mainly located in specialized microdomains called niches, where their quiescent or activated state is regulated. Abnormalities in niche function might result in inadequate myocyte formation and hence cardiac diseases. It has been demonstrated that the number of CSCs is higher in the atrial and apical myocardium than in the base-mid-region of the young and old heart [45]. Different classes of CSCs have been characterized in the adult heart and a variety of surface antigens or transcription factors have been used to define these cells (Table 1). There is not enough evidence to clarify which of these markers represent transient states of a single-cell type or belong to unrelated lineages.

c-kit receptor in the absence of common hematopoietic markers identifies a pool of resident stem cells, and it was the first marker characterizing CSCs. c-kit-positive CSCs are lineage-negative clonogenic cells that may differentiate into myocytes, vascular smooth muscle cells, and endothelial cells. Subsequently, different membrane markers (Sca-1, Abcg-2, Flk-1, and WT1) and transcription factors (Isl-1, GATA4, Nkx2.5, and Mef2c) have been employed to identify these cells.

Sca-1 (stem cell antigen-1) is another important cell surface marker for purifying the CSCs. This Sca-1^+^ population is distinct from endothelial progenitor/precursor cells since they do not express CD34, Flk-1, or Flt-1. Sca-1^+^ cells express transcriptional regulators indicative of cardiac commitment, for example, GATA-4, Mef2c, and Tef-1 [46]. Sca-1-expressing cells are present in the myocardial interstitium and could be a source of fibroblasts and adipocytes, characteristic of a fibrofatty heart condition [46].

Isl-1 is a marker of cardiac progenitor cells, mostly in the right ventricle and outflow tract, but also label of cardiac neural crest [47]. Isl-1 and GATA4 are transcriptional coactivators of the myocyte transcription factor MEF2C. The Isl-1 transcription factor is associated with the commitment to the myocyte lineage of cardiac cells and transforming them from their undifferentiated stem cell state. In the adult heart, they have only modest ability to divide, and few myocytes or vascular endothelial and smooth muscle cells originate from them [48]. The side population of CSCs is another pool of putative cardiac progenitors that form colonies in semisolid media and differentiate into cardiomyocytes [49]. They are identified by their unique ability to efflux DNA binding dyes through an ATP-binding cassette transporter. Bcrp1 is the molecular determinant of the side population phenotype. They are mainly located within the intima of the vessel wall. However, they can also be detected in the perivascular region and myocardial interstitium [50]. After injury, side population cells generate predominantly vimentin-positive fibroblasts and calponin-positive smooth muscle cells [51]. Another type of CSCs in the heart might be cardiosphere-derived cells. Cardiospheres are self-assembling cellular clusters from cardiac explants and myocardial biopsies. Cardiospheres include a core of c-kit-positive stem cells, layers of differentiating cells expressing myocyte proteins and connexin 43, and an outer surface composed of mesenchymal stromal cells. They contain promotive and early committed progenitor cells for cardiomyocytes, endothelial cells, and smooth muscle cells, and they represent an attractive cell source for cardiac regeneration [52].

MSCs make up another class of multipotent cells that probably have a proepicardial origin and occupy perivascular niches [53]. Their characterization is complex, since MSC subpopulations may express different surface molecules and phenotypic characteristics of endothelial, smooth muscle, skeletal, and cardiac muscle cells. So far, it has not been completely clarified to what extent differences in these surface markers represent separate stem cell populations with different functional capacities.

Finally, it appears that, under pathological conditions, stem cells from extracardiac sources might home in the heart. It has been shown that an increase in preload results in the mobilization of progenitor cells from the bone marrow for use in neovascularization, which plays a pivotal role in cardiac hypertrophy [32].

## 5. Left Ventricular Hypertrophy in Aortic Diseases

In the heart, increased afterload leads to the upregulation of EPCs from the bone marrow, which may contribute to myocardial angiogenesis and remodeling [32]. Chronic increased left ventricular afterload, such as in aortic stenosis, leads to cardiac remodeling in the form of concentric LVH, which is characterized by an increase in the number and hypertrophy of myocardial cells, the activation and proliferation of fibroblasts, and increased fibrosis in the myocardium. Pressure overload, mediated by aortic stenosis, may induce cardiomyocyte regeneration. Specifically, Urbanek et al. have shown that in human aortic stenosis the increase in cardiac mass is the result of both hypertrophy of existing myocytes and hyperplasia caused by the differentiation of stem-like cells to cardiogenic and myocyte precursors [54]. However, apart from progenitor cells that are resident in the myocardium, others may be recruited from noncardiac sources, from which new myocytes can be derived. Their number increases by more than 13-fold in the hypertrophic myocardium of patients with aortic stenosis. In addition, patients with aortic stenosis were found to have increased caspase-3 activity and reduced telomere-repeating factor-2 expression in EPCs compared with controls, suggesting increased cell senescence as well as enhanced apoptosis of EPCs in those patients [55].

In addition, studies of aortic regurgitation, which is characterized mainly by volume overload, have also found activation of EPCs in numbers that were correlated with LVH [56].

Increased numbers of MSCs were also found in biopsies from patients undergoing aortic valve replacement, accompanied by a heightened capacity for differentiation [57]. In addition, EPCs are mobilized and homed into the myocardium following transverse aortic constriction in mice [58]. However, this capability appears to be lost with aging [44].

## 6. Left Ventricular Hypertrophy in Arterial Hypertension

The participation of progenitor cells in arterial hypertension started to become apparent from studies in animal models. Bone marrow seems to be an important organ that participates in the pathophysiology of cardiac remodeling in hypertension. Experimental studies have shown that an increase in preload results in the mobilization of progenitor cells from the bone marrow and migration into the heart, which plays an important role in cardiac hypertrophy [43]. There are indications that the recruitment of bone marrow-derived cells is involved in cardiac myocyte hypertrophy and maintenance of function in response to pressure overload [43]. A previous study from our department has shown increased expression of myocardin and GATA4 genes in the peripheral blood mononuclear cell fraction of hypertensive patients, implying the presence of mesenchymal progenitor cells in the peripheral blood that could possibly be intended to differentiate into cells of the cardiac series [59]. In this study, myocardin and GATA4 expression was associated with both blood pressure levels and LVH in a hypertensive population.

MSCs have a highly plastic differentiation potential that results in adipogenesis, osteogenesis, and chondrogenesis, as well as endothelial, cardiovascular, and neovascular differentiation. Bone marrow-derived MSCs have been proven to generate functional cardiomyocytes [60]. These are present as a rare population of cells in bone marrow, but under pathological conditions a small percentage of them are mobilized and may also be detected in peripheral blood. Further to the previous study, our research group has also determined that patients with essential hypertension have higher levels of circulating MSCs compared to normotensives, while the number of MSCs correlates with left ventricular mass index, suggesting that they might be implicated in the pathophysiology of hypertensive cardiomyopathy [61].

Many investigators have already demonstrated that arterial hypertension is associated with various changes in the number and function of subpopulations of progenitor cells, especially those in peripheral blood. Recently published data indicate an association between hypertension with LVH and changes in circulating proangiogenic hematopoietic cell numbers and/or phenotypes [32]. In the same study, NOX2, MnSOD, CAT, and GPx-1 were overexpressed in CD34^+^ cells from hypertensives with increased arterial stiffness. EPC-mediated angiogenesis in pressure overload also depends on NO production, which plays an important role in stem cell-induced cardiac remodeling [62]. Recent animal studies showed that eNOS affects almost all stages of the process, from the production of these cells to their mobilization and migratory capacity [63].

On the other hand, other studies have shown that patients with essential hypertension and electrocardiographic evidence of LVH have fewer circulating EPCs with reduced adhesive function compared to patients without LVH [64]. In addition, EPCs might play a beneficial role in angiotensin-induced cardiac hypertrophy, by releasing cellular microvesicles that deliver protective gene messages to cardiac myocytes [65]. Furthermore, angiotensin participates in the process of myocardial remodeling and hypertrophy; experimental studies have shown that angiotensin infusion in animal models results in cardiac hypertrophy and fibrosis by mobilization and differentiation of a CD34^+^/CD45^+^ fibroblast precursor population [66].

Notably, animal studies have shown that intrarenal delivery of EPCs or MSCs attenuates renovascular hypertension-induced myocardial injury. MSCs restore diastolic function more effectively than EPCs in myocardium of pigs with renovascular hypertension [67]. However, their pathophysiological role has not been clarified and different studies have produced conflicting results. It is likely that their role in cardiac remodeling is not always beneficial, or only up to a point. Clarification of these matters would allow us to better evaluate the prospects of therapeutic applications in the future.

## 7. Stem Cells and Hypertrophic Cardiomyopathy

Hypertrophic cardiomyopathy (HCM) is known to be dependent on genetic causes. However, the mechanisms by which sarcomeric gene mutations lead to pathological myocardial hypertrophy have not been fully elucidated.

Animal studies have demonstrated the presence of an increased number of c-kit-positive, MDR-positive, and Sca-1-positive stem cells within the myocardium of hereditary delta-SG null hamsters, a spontaneously occurring model of hypertrophic cardiomyopathy [68]. In addition, ablation of GSK-3*β* led to impaired cardiomyocyte differentiation in ES cells and to pronounced hyperplasia of cardiomyocytes during embryonic development [69]. Lan et al. generated human-induced pluripotent stem cell-derived cardiomyocytes (iPSC-CMs) from a family carrying an autosomal dominant missense mutation on exon 18 of the MYH7 gene [70]. They demonstrated that iPSC-CMs can recapitulate the hypertrophic cardiomyopathy phenotype at the single-cell level, including cellular hypertrophy, the calcineurin/nuclear factor of activated T-cell, upregulation of hypertrophic transcription factors, and arrhythmia.

Notably, an altered expression of early cardiac marker genes and differentiation-specific marker genes was found in the peripheral blood mononuclear cell fraction of patients with HCM compared to control individuals [71]. Additionally, patients with HCM show an increased mobilization of MSCs compared to healthy individuals [61].

Although further research is needed to reveal the clinical significance of these findings, these data open a new dimension in the pathophysiology of HCM and may hold out the hope of future therapeutic possibilities.

## 8. The Regulatory Role of miRNAs in Stem Cell Function

miRNAs, small RNAs that regulate gene expression in the cell by binding to mRNAs, appear to play an important regulatory role in the differentiation of stem cells and their participation in myocardial hypertrophy. Many miRNAs, such as miR-371–373 and miR-17–92, have a different expression in stem cells compared to more differentiated cells [72]. For some miRNAs, we understand their way of action in more detail; for example, miR-290 helps maintain the stem cell state by targeting the NF-*κ*B subunit p65, which is known to promote differentiation [73]. There are also ample data concerning miR-1 and miR-133a, which are highly expressed in the adult heart and stimulate myofibroblast proliferation. However, their role in EPCs seems to be an antagonistic one, since miR-1 promotes the differentiation of ESCs into cardiac cells by inhibiting the notch delta-like ligand, while miR-133 inhibits their differentiation into cardiac muscle [74].

Stem cells have the capacity to self-renew and regenerate throughout their lifetime. Apart from playing an important role in cardiac development, miRNAs also play a key role in stem cell renewal. Changes in their expression in progenitor cells might attenuate cell migration and proliferation.

In cardiac hypertrophy, several miRNAs, such as miR-208, miR-21, miR-125, miR-129, and miR-195, are increased, whereas others, such as miR-1 and miR-133, are decreased [75]. miR-499, which is abundant in cardiomyocytes and essentially absent in CSCs, can be transferred from myocytes to resident stem cells* via* gap junction channels, resulting in the enhanced differentiation of the primitive cells toward myocytic lineage [76]. CSCs start to express miR-499, and the quantity increases as the differentiation advances. miR-499 in rat bone marrow MSCs induces them toward cardiac differentiation by activating the WNT/*β*-catenin signal pathway [77]. In addition, overexpression of miR-499 and miR-1 resulted in upregulation of important cardiac myosin heavy-chain genes in embryoid bodies that are involved in the cardiac specification of human embryonic stem cells [78] and regulate the proliferation of human CSCs and their differentiation into cardiomyocytes [79]. miR-126 expression contributes to the impairment of the regenerative capabilities of proangiogenic cells in patients with diabetes mellitus [80]. miR-99b, miR-181a, and miR-181b can potentiate differentiation in endothelial cells from hESCs [81]. Combining miR-modulation with stem cell therapy might be a potential future therapeutic strategy.

## 9. Stem Cells and New Perspectives

The participation of stem and progenitor cells in myocardial regeneration by giving rise to new myocytes and vascular structures could open the door to new therapeutic possibilities in the future. Some studies have been performed with this in view, mainly with regard to heart failure or myocardial infarction. Stem cell-based therapies are a promising intervention for the treatment of heart failure secondary to ischemic and nonischemic cardiomyopathy. The REPAIR-AMI trial has shown that transfer of bone marrow-derived stem cells to the myocardium may improve global function and remodeling [82], while newer studies found that they can intervene beneficially in maladaptive hypertrophy, especially after myocardial infarction [83]. A large meta-analysis, including forty-eight randomized controlled trials, indicated that implantation of bone marrow stem cells has a therapeutic potential in ischemic cardiomyopathy, since it improves ejection fraction, reduces infarct size, and ameliorates adverse ventricular remodeling [84]. Similar results emerged from another recent meta-analysis, indicating that autologous cell therapy may be beneficial for patients having heart failure [85].

However, the literature contains studies with conflicting results, probably because our knowledge of the mechanisms is still limited. The properties of stem cells offer the prospect of cell therapy to prevent adverse remodeling. Thus, in theory, if we could fully understand the pathophysiology of pathological myocardial hypertrophy, we might be able to intervene in the mechanisms, accentuating their beneficial role and attenuating the detrimental effects. In addition, clinical heterogeneity can confuse the results in myocardial cell regeneration research.

All these developments are still under investigation but hold the promise of potential adjunctive therapies in the future, provided that the encouraging initial results are confirmed in large clinical trials [86].

## 10. Conclusion

Stem and progenitor cells contribute to the renewal of adult mammalian cardiomyocytes in cases of myocardial injury, such as myocardial infarction or pressure or volume overload. They are involved in cardiac myocyte hypertrophy and homeostasis, activated in pathological LVH, and play a role in myocardial repair. In addition, precursor migratory cells participate in the formation of almost all cardiac structures in myocardial hypertrophy. Nonetheless, the pathophysiological mechanisms are still obscure, and further experimental and clinical studies are required. The properties of stem cells suggest future prospects for regenerative cell therapy to prevent adverse remodeling.

## Figures and Tables

**Table 1:** Classes of cardiac stem/progenitor cells in the adult heart.

Class of cardiac stem/progenitor cells	Characterization
Cardiac stem cells	c-kit, Sca-1, Abcg-2, Flk-1, and WT1
Side population of cardiac stem cells	Bcrp1
Mesenchymal stem cells	CD70^+^, CD90^+^, CD105^+^, and CD34^−^
Cardiosphere-derived cells	Cellular clusters from cardiac explants
